# Modeling Mechanisms of In Vivo Variability in Methotrexate Accumulation and Folate Pathway Inhibition in Acute Lymphoblastic Leukemia Cells

**DOI:** 10.1371/journal.pcbi.1001019

**Published:** 2010-12-02

**Authors:** John C. Panetta, Alex Sparreboom, Ching-Hon Pui, Mary V. Relling, William E. Evans

**Affiliations:** 1 Department of Pharmaceutical Sciences, St. Jude Children's Research Hospital, Memphis, Tennessee, United States of America; 2 Colleges of Medicine and Pharmacy, University of Tennessee, Memphis, Tennessee, United States of America; 3 Department of Oncology, St. Jude Children's Research Hospital, Memphis, Tennessee, United States of America; University of Virginia, United States of America

## Abstract

Methotrexate (MTX) is widely used for the treatment of childhood acute lymphoblastic leukemia (ALL). The accumulation of MTX and its active metabolites, methotrexate polyglutamates (MTXPG), in ALL cells is an important determinant of its antileukemic effects. We studied 194 of 356 patients enrolled on St. Jude Total XV protocol for newly diagnosed ALL with the goal of characterizing the intracellular pharmacokinetics of MTXPG in leukemia cells; relating these pharmacokinetics to ALL lineage, ploidy and molecular subtype; and using a folate pathway model to simulate optimal treatment strategies. Serial MTX concentrations were measured in plasma and intracellular MTXPG concentrations were measured in circulating leukemia cells. A pharmacokinetic model was developed which accounted for the plasma disposition of MTX along with the transport and metabolism of MTXPG. In addition, a folate pathway model was adapted to simulate the effects of treatment strategies on the inhibition of *de novo* purine synthesis (DNPS). The intracellular MTXPG pharmacokinetic model parameters differed significantly by lineage, ploidy, and molecular subtypes of ALL. Folylpolyglutamate synthetase (FPGS) activity was higher in B vs T lineage ALL (p<0.005), MTX influx and FPGS activity were higher in hyperdiploid vs non-hyperdiploid ALL (p<0.03), MTX influx and FPGS activity were lower in the t(12;21) (*ETV6-RUNX1*) subtype (p<0.05), and the ratio of FPGS to γ-glutamyl hydrolase (GGH) activity was lower in the t(1;19) (*TCF3-PBX1*) subtype (p<0.03) than other genetic subtypes. In addition, the folate pathway model showed differential inhibition of DNPS relative to MTXPG accumulation, MTX dose, and schedule. This study has provided new insights into the intracellular disposition of MTX in leukemia cells and how it affects treatment efficacy.

## Author Summary

One of the primary agents used in the treatment of childhood acute lymphoblastic leukemia (ALL) is methotrexate (MTX). By better understanding its intracellular disposition, we are able to better design treatments that circumvent drug resistance and thus help improve ALL cure rates. In this study, we develop a system of mathematical models that describe the intracellular disposition of MTX along with its inhibition of important biosynthetic pathways necessary for cell division. First, we used the models to describe the disposition of intracellular MTX in a cohort of 194 patients enrolled on St. Jude Total XV protocol for newly diagnosed ALL. The results of this modeling allowed us to determine mechanisms of *in vivo* variability in MTX accumulation. These mechanisms related to both the influx and efflux of the drug along with the enzymes related to its metabolism. Next, we used model simulations to show the effects of changes in MTX dose and schedule on its efficacy. The results of these simulations show that longer infusions yield better efficacy and that higher MTX doses can circumvent resistance observed in ALL subtypes with lower intracellular MTX accumulate. The results from this study provide new insights into the design of more effective therapy for pediatric ALL.

## Introduction

Methotrexate (MTX) is one of the primary anticancer agents used for the treatment of acute lymphoblastic leukemia (ALL) [1]–[3]. The ability of cells to accumulate intracellular polyglutamate metabolites of MTX (MTXPG) is an important factor in its antileukemic effects [4]. Specifically, MTXPG inhibits the folate pathway by competitively inhibiting several important enzymes including: dihydrofolate reductase (DHFR), thymidylate synthase (TS), glycinamide ribonucleotide transformylase (GART), and aminoimidazole carboxamide ribonucleotide transformylase (AICART). This inhibition leads to reduced or blocked TS and de novo purine synthesis (DNPS), which are needed for DNA synthesis. There is large variability in MTXPG accumulation and a variety of studies have related differences in its accumulation to ALL lineage, ploidy, molecular subtype, and folate pathway gene expression [5]–[8]. Thus, developing a better understanding of the underlying mechanisms responsible for these differences in cellular disposition of MTX is important for understanding the basis for inter-patient differences in MTX's antileukemic effects and to identify strategies to circumvent mechanisms of resistance.

Pharmacokinetic and pharmacodynamic modeling is a useful approach to quantify the intracellular kinetics of MTX and to aid in understanding the underlying mechanisms related to differences in MTXPG accumulation [9]. For example, modeling can be helpful in addressing whether higher accumulation of intracellular MTXPG is related to higher formation of polyglutamates via higher folylpolyglutamate synthetase (FPGS) activity, lower degradation of polyglutamates via γ-glutamyl hydrolase (GGH), or differences in MTX influx or efflux from leukemic cells, In addition, there are numerous models describing MTX inhibition of target enzymes in the folate pathway [10]–[18], which can be exploited to advance our understanding of how folate inhibitors such as MTX alter folate homeostasis leading to its antileukemic effects.

In an effort to better understand the underlying dynamics of the observed differences in MTXPG accumulation along with their differential effects on folate kinetics, we used a pharmacokinetic model to characterize the disposition of plasma MTX and intracellular MTXPG [9] along with a pharmacodynamic model to describe the dynamics of perturbations in the folate pathway [12]. These models allowed us to relate differential disposition of intracellular MTXPG to changes in transport of MTX into and out of leukemic blasts along with metabolism of intracellular MTXPG. In addition, the folate pathway model allowed us to investigate how this differential disposition of intracellular MTXPG alters folate homeostasis and its downstream consequences.

Therefore, the objectives of this study were to determine the intracellular pharmacokinetics of MTXPG in circulating leukemic blasts, and to assess the relationship between these pharmacokinetic parameters and covariates including ALL lineage, ploidy, molecular subtype, and gene expression of and polymorphisms in or flanking genes related to MTX transport and metabolism. In addition, we analyzed the effects of intracellular MTXPG disposition, MTX dose, and MTX infusion schedule on the folate pathway.

## Results

This study included 194 patients with newly diagnosed ALL who were enrolled on the St. Jude Total XV protocol and had sufficient circulating ALL cells to permit serial measurement of MTXPG in their leukemia cells. There were no differences in demographics, lineage, ploidy, or molecular subtype between the 194 patients and all other patients on the Total XV protocol (n = 162). Not surprisingly, diagnostic WBC counts were higher in the 194 patients than those in all other patients due to the need for sufficient circulating ALL cells to perform the MTXPG assay (**Table S1**). A summary of the demographic, lineage, chromosomal ploidy, molecular subtype, and randomized window therapy arm for the patients included in this study are shown in **Table 1**.

**Table 1:** Summary statistics of patients in this study.

	Randomization Arm	Window Therapy	
		1 g/m^2^ MTX infused IV over 24 hrs	1 g/m^2^ MTX infused IV over 4 hrs
**Number of peripheral blast samples (n)**		99	95
**Sex**	Male	47	55
	Female	52	40
**Self-Declared Race**	Caucasian	83	71
	African American	12	18
	Asian	2	2
	Other	2	4
**Lineage/Ploidy/Molecular Subtype**	B lineage Hyperdiploid	31	29
	B lineage Non-Hyperdiploid	28	24
	t(12;21)[*ETV6-RUNX1*]	24	22
	t(1;19)[*TCF3-PBX1*]	6	8
	T lineage	10	12

### Methotrexate Plasma and Leukemia Cell Intracellular Pharmacokinetics

**Figure 1:**
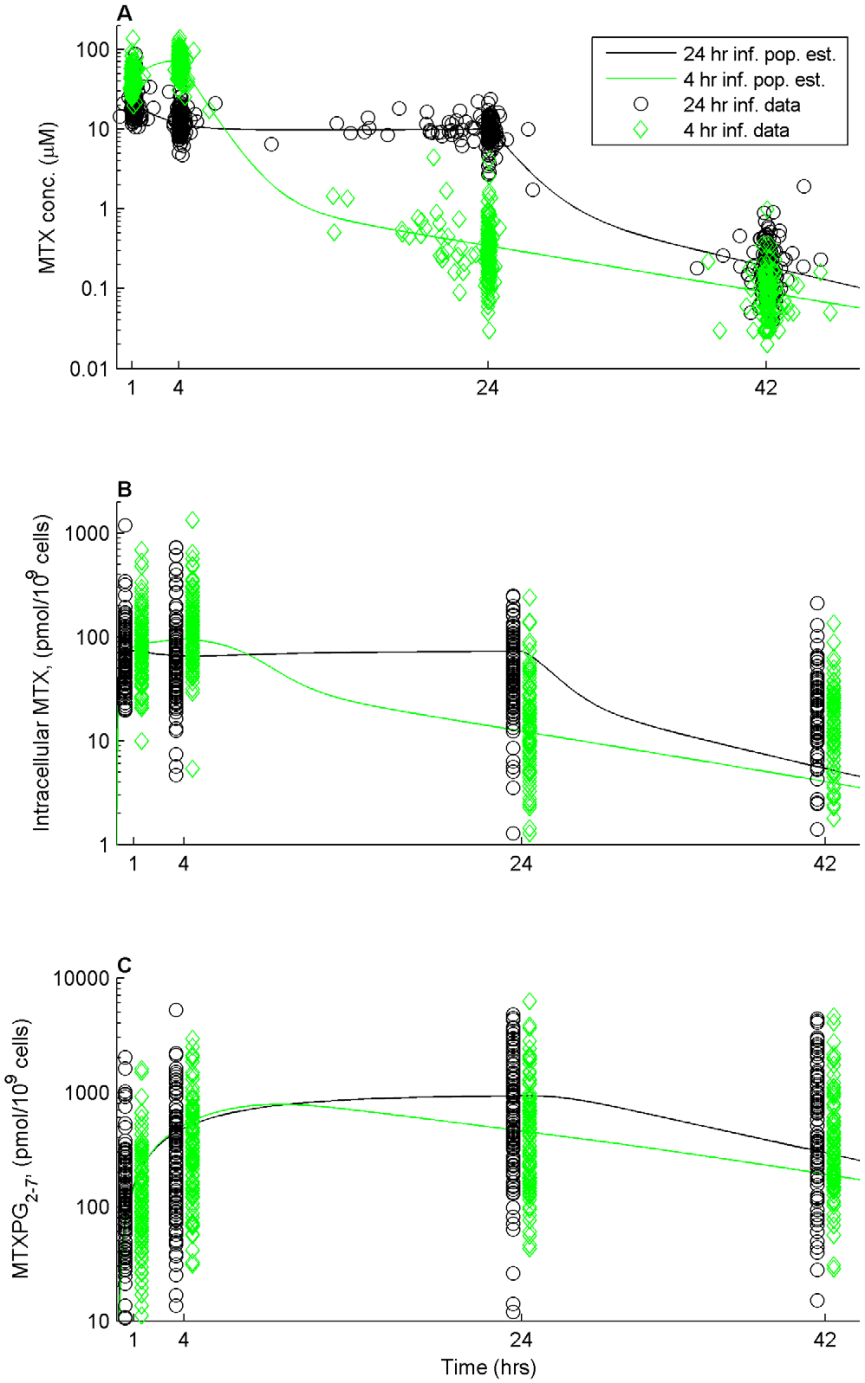
MTX and MTXPG concentration vs time plots. (**A**) Plasma MTX concentration (µM) vs time (hrs). (**B**) Intracellular MTX (MTXPG_1_) concentration in peripheral blood cells (pmol/10^9^ cells) vs time (hrs). (**C**) Intracellular MTXPG_2-7_ concentration in peripheral blood cells (pmol/10^9^ cells) vs time (hrs). Black lines and circles represent the model fit and data given a 24 hour infusion and green lines and dimonds represent the model fit and data given a 4 hour infusion. We plotted the MTXPG concentrations at fixed time points around each sampling time to prevent the samples from the two different infusion groups from overlapping.

A total of 791 plasma samples in 194 patients were assayed to determine the plasma MTX disposition. **Figure 1A** shows the concentration vs time plot of these data along with the population average model fit of the data sub-grouped by infusion length. The median clearance of MTX was higher in the 24 hr infusion group compared to the 4 hr infusion group (122.6 ml/min/m^2^ vs 108.6 ml/min/m^2^; p<0.001).

A total of 732 peripheral blood leukemia cell samples in 194 patients were assayed for intracellular MTXPG disposition. Fixing the plasma MTX pharmacokinetic parameters to each individual's estimates, the intracellular population pharmacokinetic parameters for MTXPG were determined and the descriptive statistics of the individual estimates (conditional means) are shown in **Table 2**. In addition, the population estimates, relative standard error estimates of the population estimates, inter-individual variability estimates, and sensitivity analysis of the individual estimates are summarized in **Table S2**. **Figure 1** shows the concentration vs time plot of intracellular MTX (or MTXPG_1_) (**Figure 1B**) and total intracellular MTXPG_2-7_ (**Figure 1C**) along with the population average model fit (for non-hyperdiploid B-lineage ALL) of the data. In addition, several representative plots of individual model fits to the data are shown in **Figure S1**.

**Table 2:** MTX and MTXPG model parameters.

Parameter	BHD (n = 60)	BNHD (n = 52)	ETV6-RUNX1 (n = 46)	TCF3-PBX1 (n = 14)	T (n = 22)	Subtype	p-val^b^Lineage	Ploidy
**CL** (ml/min/m^2^)	120.51 (56.43, 173.00)	116.15 (35.67, 191.17)	120.36 (40.02, 193.50)	117.07 (55.50, 252.17)	101.02 (39.35, 217.67)	0.06	<0.02	0.06
**V** (L/m^2^)	13.10 (4.20, 18.43)	11.19 (4.59, 18.25)	11.48 (4.75, 17.74)	11.50 (5.82, 17.12)	10.76 (2.90, 15.86)	>0.1	0.06	<0.03
**k_12_** (1/hr)	0.049 (0.010, 0.12)	0.050 (0.0066, 0.14)	0.033 (0.011, 0.12)	0.028 (0.011, 0.10)	0.046 (0.010, 0.071)	<0.002	>0.1	<0.02
**k_21_** (1/hr)	0.11 (0.036, 0.15)	0.11 (0.0040, 0.13)	0.11 (0.036, 0.13)	0.11 (0.028, 0.12)	0.11 (0.048, 0.12)	>0.1	>0.1	>0.1
**V_max-in_** (pmol/10^9^ cells/h)	1439.03 (349.10, 8075.77)	830.83 (103.14, 11030.30)	989.57 (85.75, 6663.96)	838.78 (60.95, 5224.35)	879.92 (199.77, 26364.30)	0.08	>0.1	<0.006
**K_m-in_** (µM)	3.54 (0.03, 767.20)	5.03 (0.03, 107.21)	1.41 (0.01, 174.68)	2.07 (0.15, 83.64)	2.44 (0.25, 87.10)	>0.1	>0.1	>0.1
**Influx** (250^−1^/h)^a^	344.23 (1.91, 68237.70)	215.4 (6.41, 86310.07)	325.26 (3.08, 116330.25)	466.69 (0.98, 12858.28)	286.93 (13.33, 3847.66)	>0.1	>0.1	>0.1
**K_efflux_** (1/h)	6.58 (0.71, 148.49)	6.07 (0.53, 124.88)	8.37 (0.24, 76.71)	10.04 (0.12, 81.63)	6.39 (1.27, 98.27)	>0.1	>0.1	>0.1
**NET-influx** (pmol/10^9^ cells)	213.69 (25.77, 1158.66)	105.21 (16.02, 1233.83)	90.44 (24.78, 1539.49)	156.98 (26.23, 674.26)	130.63 (41.27, 421.11)	<0.02	>0.1	<0.0009
**V_max-fpgs_** (pmol/10^9^ cells/h)	453.61 (24.23, 5601.34)	360.62 (43.12, 3030.52)	250.97 (58.02, 3384.47)	235.38 (75.02, 1068.70)	160.24 (14.62, 1364.28)	<0.0002	<0.0002	<0.005
**K_m-fpgs_** (pmol/10^9^ cells)	24.58 (0.17, 49068.40)	44.11 (3.61, 1178.71)	38.68 (0.80, 1524.17)	33.06 (2.33, 1689.72)	35.88 (3.21, 378.80)	>0.1	>0.1	>0.1
**FPGS** (1/h)	14.57 (0.11, 146.20)	7.63 (0.20, 329.22)	8.13 (0.17, 107.35)	5.76 (0.30, 70.27)	4.51 (1.35, 21.21)	<0.005	<0.01	<0.001
**K_ggh_** (1/h)	0.19 (0.04, 3.66)	0.13 (0.03, 4.81)	0.24 (0.02, 2.67)	0.33 (0.07, 1.57)	0.15 (0.04, 0.61)	>0.1	>0.1	>0.1
**NET-PG** (pmol/10^9^ cells)	2052.85 (58.89, 109504.90)	2451.61 (102.66, 19501.03)	1293.4 (115.98, 16879.21)	733.48 (145.00, 6794.54)	1014.49 (247.65, 3524.49)	<0.003	<0.05	>0.1

a Based on 250 fl/cell.

b Kruskal-Wallis ANOVA.

Descriptive statistics, median (minimum, maximum), of the individual MTX and MTXPG pharmacokinetic parameters subdivided by molecular subtype. p-values are shown for comparisons by molecular subtype, lineage, and chromosomal ploidy.

### Covariate Analysis

It has been previously reported that there are significant differences in intracellular MTXPG accumulation by ALL lineage, ploidy, and molecular subtype [4], [5], [19]. Using the pharmacokinetic model of the intracellular disposition in peripheral blasts of MTXPG, we quantified how differences in MTXPG disposition related to the model estimated parameters describing MTXPG influx, efflux, FPGS, and GGH activity.

### MTX Influx and Efflux

**Figure 2:**
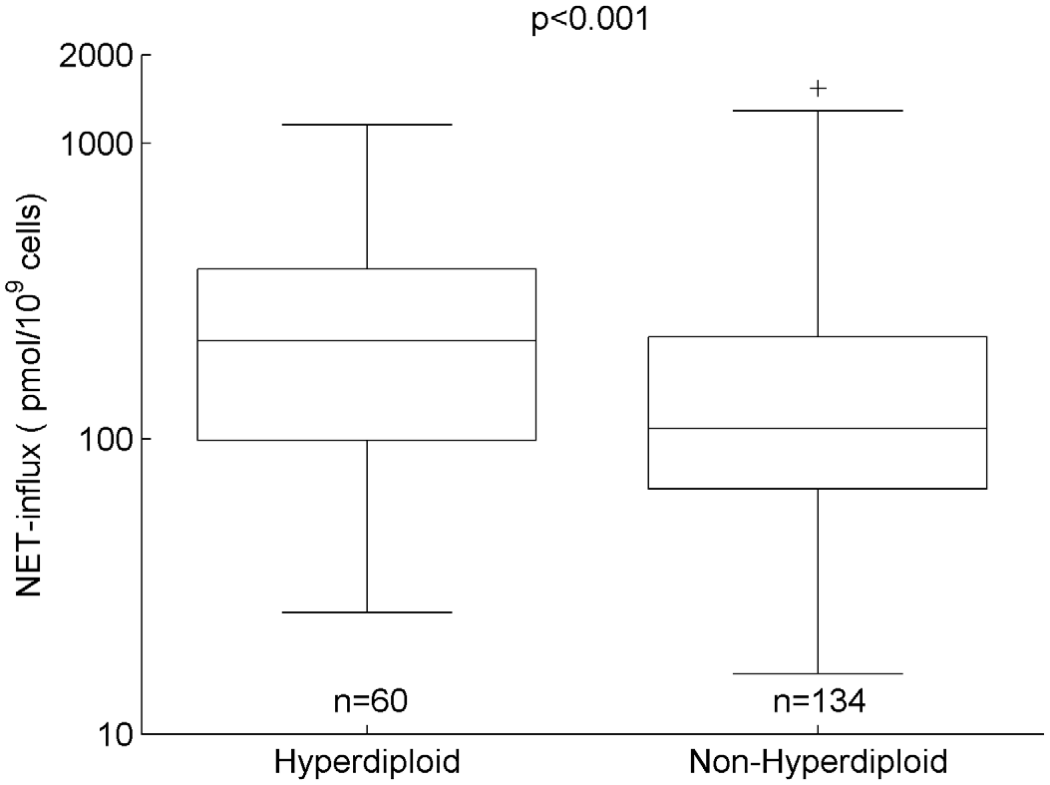
MTX net influx vs ALL ploidy. NET-influx (model parameter describing the net influx of MTX) vs ALL ploidy (hyperdiploid vs non-hyperdiploid). Median, quartiles, non-outliers range (defined as 1.5 times the interquartile range), and outliers (plus-marks) are depicted for B-lineage ALL.

ALL chromosomal ploidy exhibited differences in influx and efflux parameters for MTX. Specifically, NET-influx was 2 times higher (p<0.0009) in hyperdiploid ALL compared to non-hyperdiploid ALL (**Figure 2**). In addition, we observed higher efflux (1.8 times higher; p<0.003) and lower NET-influx (1.5 times lower; p<0.02) in patients randomized to the 24 hr infusion compared to the 4 hr infusion.

### MTXPG Glutamylation and Degradation via Hydrolysis

**Figure 3:**
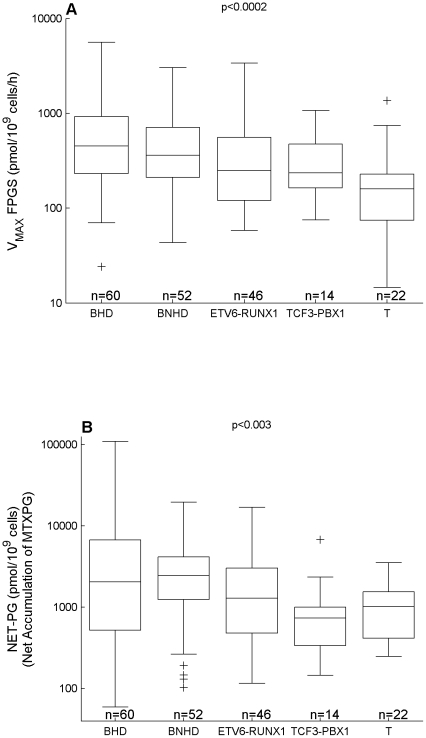
MTX metabolizing enzyme model parameters vs ALL subtype. (**A**) V_MAX_ FPGS vs molecular subtype. (**B**) NET-PG (net accumulation of MTXPG) vs molecular subtype. Median, quartiles, non-outliers range (defined as 1.5 times the interquartile range), and outliers (plus-marks) are depicted. The subtypes are defined as follows: BHD, B-lineage hyperdiploid ALL; BNHD, B-lineage non-hyperdiploid without the t(12;21) [*ETV6-RUNX1*] or t(1;19) [*TCF3-PBX1*] translocation; ETV6-RUNX1, B-lineage non-hyperdiploid with the t(12;21) [*ETV6-RUNX1*] translocation; TCF3-PBX1, B-lineage non-hyperdiploid with the t(1;19) [*TCF3-PBX1*] translocation; and T, T-lineage ALL.

The model parameters describing FPGS activity differed significantly by ALL lineage and molecular subtype. Specifically, the maximum FPGS activity was 2.1 times higher (p<0.0002) in B-lineage ALL compared to T-lineage ALL. In addition, there was a significant difference (p<0.0002) in the maximum FPGS activity among the different molecular subtypes of ALL with the highest activity in B-lineage hyperdiploid ALL followed in decreasing order by B-lineage non-hyperdiploid, t(12;21) [*ETV6-RUNX1*], t(1;19) [*TCF3-PBX1*], and T-lineage ALL (**Figure 3A**). These differences translated to differential net accumulation (p<0.003) of MTXPG (NET-PG) with highest accumulation in B-lineage hyperdiploid and B-lineage non-hyperdiploid ALL, followed by t(12;21) [*ETV6-RUNX1*], T-lineage, and t(1;19) [*TCF3-PBX1*] ALL (**Figure 3B**).

### Gene Expression and Polymorphisms

**Figure 4:**
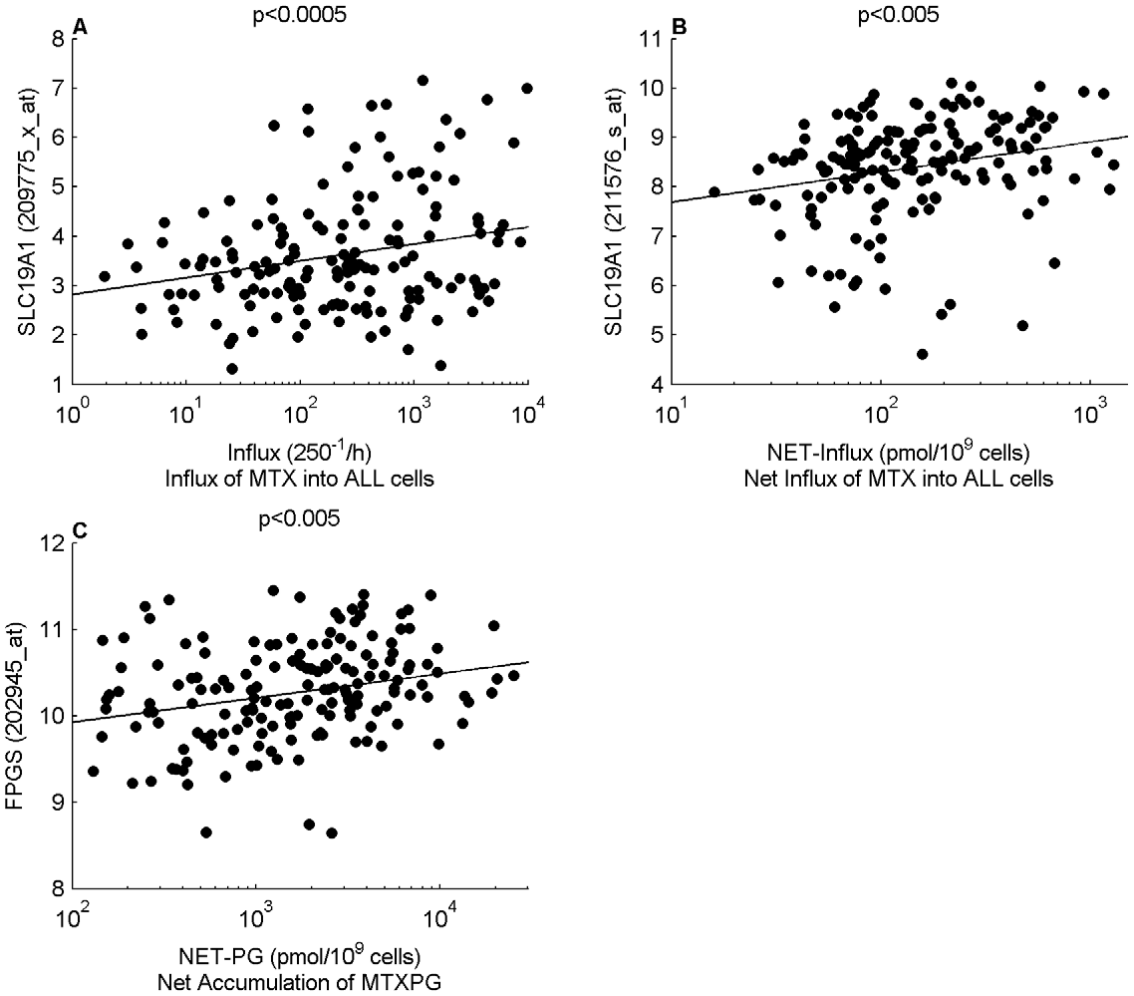
MTXPG model parameters vs gene expression in ALL blasts at diagnosis. (**A**) SLC19A1 expression (Probe Set ID: 209775_x_at) vs Influx. (**B**) SLC19A1 (Probe Set ID: 211576_s_at) vs NET-influx. (**C**) FPGS expression (Probe Set ID: 202945_at) vs NET-PG.

**Figure 5:**
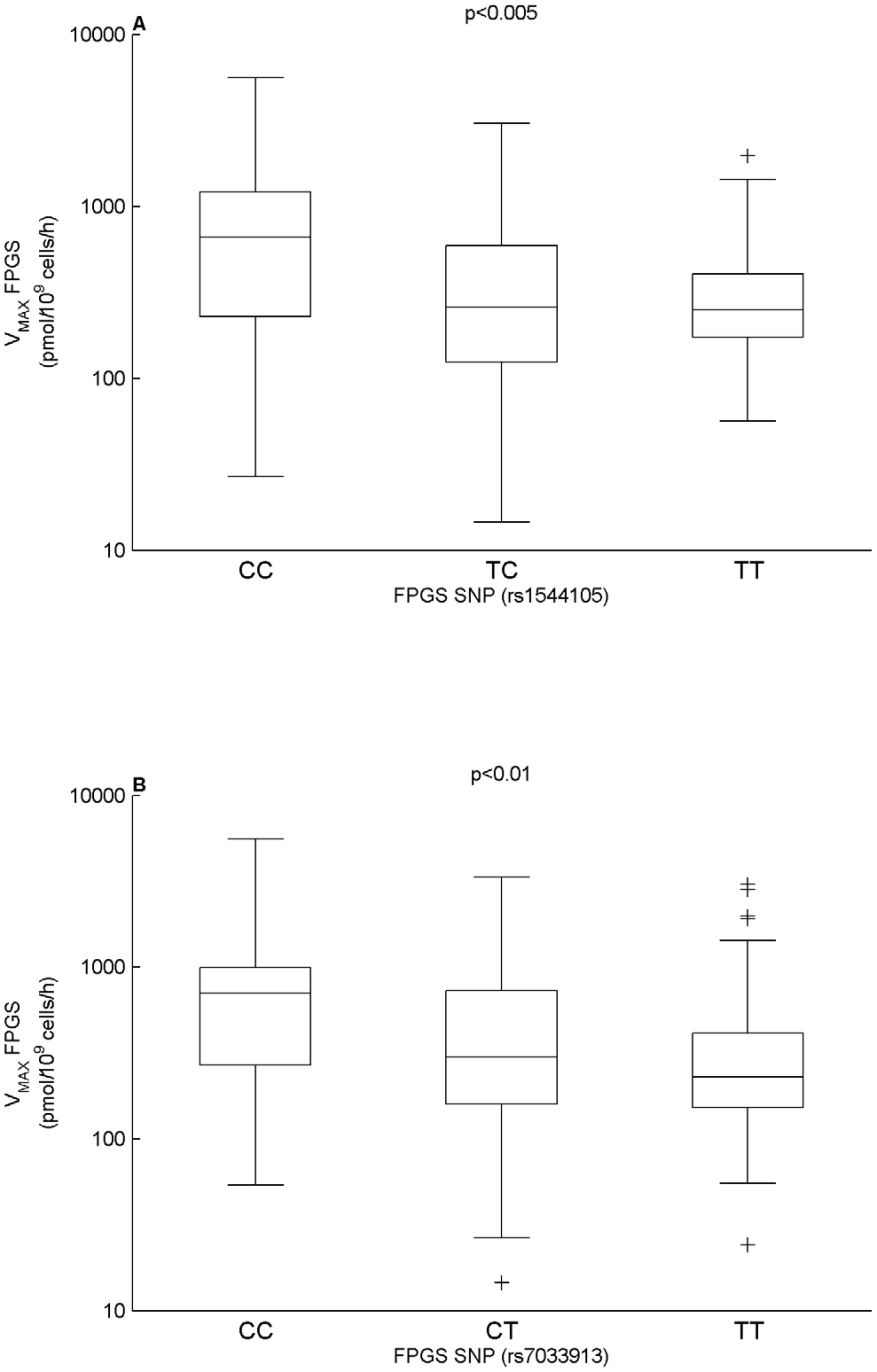
MTXPG model parameters vs germline SNPs. (**A**) V_MAX_FPGS vs FPGS SNP (DB SNP ID: rs1544105). (**B**) V_MAX_FPGS vs FPGS SNP (DB SNP ID: rs7033913). The p-values were determined by the Kruskal-Wallis ANOVA.

We also investigated how the MTXPG model parameters related to gene expression (mRNA) in ALL cells and germline polymorphisms in or flanking genes related to MTX transport and metabolism. These data were available for 168 and 190 of the patients, respectively. First, we assessed how MTX transporter gene expression and polymorphisms related to the model estimated parameters for MTX influx and efflux. Specifically, MTX influx (*V_max-in_/K_m-in_*) increased as the expression of SLC19A1 (probe set ID: 209775_x_at) increased (p<0.0005) and NET-influx increased as the expression of SLC19A1 (probe set ID: 211576_s_at) increased (p<0.005) (**Figure 4A–B**). None of the polymorphisms in or flanking transporter genes that we evaluated were significantly related to the MTX influx or efflux parameters. Next, we studied how FPGS and GGH gene expression and polymorphisms related to the model estimated parameters for FPGS and GGH activity. Specifically, net accumulation of MTXPG (NET-PG) increased as the expression of FPGS (probe set ID: 202945_at) increased (p<0.005) (**Figure 4C**). In addition, two SNPs upstream of FPGS (DB SNP ID: rs1544105, 2782 base pairs (bp) upstream and DB SNP ID: rs7033913, 4440 bp upstream) showed a significant relation to maximum FPGS activity (CC 2.6 times higher activity compared to TT: p<0.005; CC 2.4 times higher activity compared to TT: p<0.01, respectively) (**Figure 5A–B**).

### Folate Pathway Simulations

**Figure 6:**
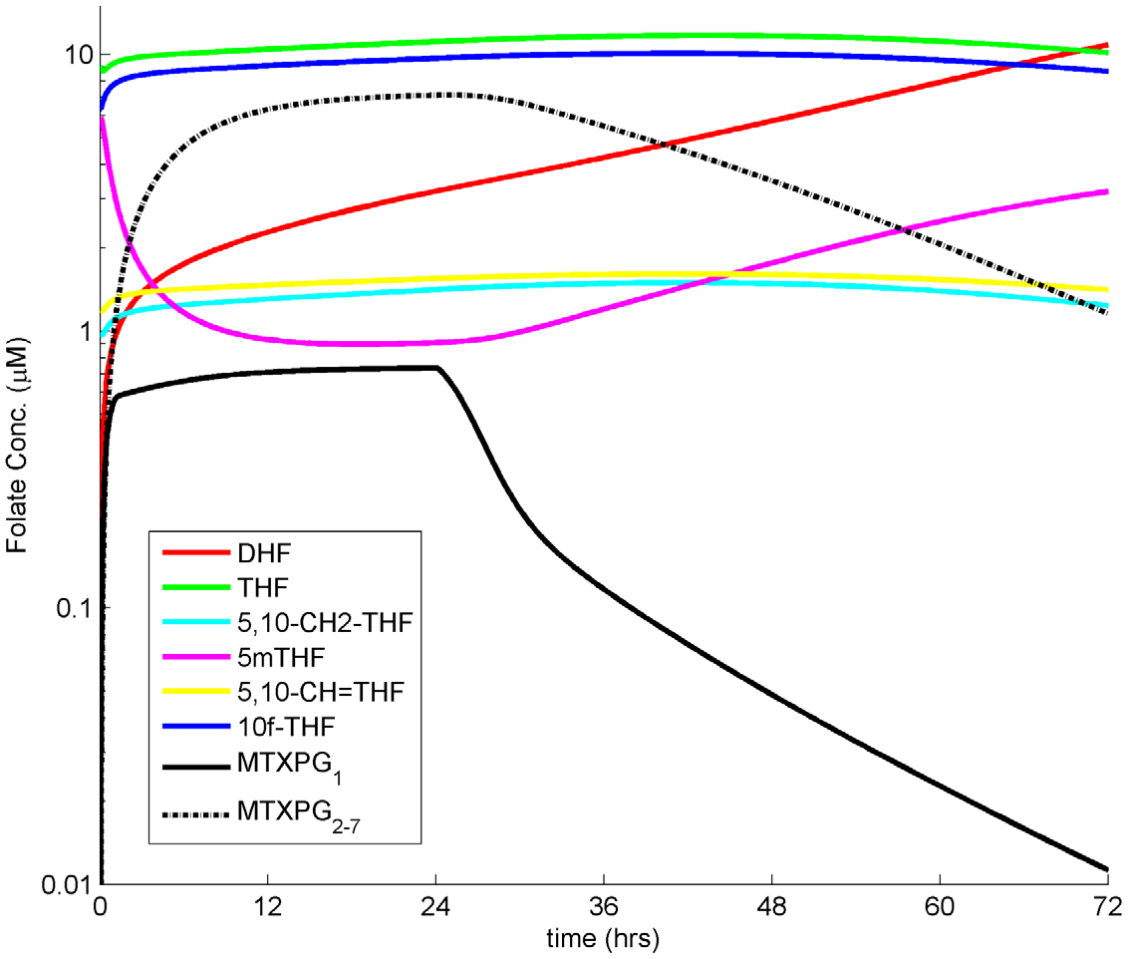
Individual simulated intracellular MTXPG and intracellular folate concentrations vs time. Dose: 1 g/m^2^; Infusion time: 24 hrs. Red: DHF; Green: THF; Light Blue: 5,10-CH2-THF; Magenta: 5mTHF; Yellow: 5,10-CH = THF; Dark Blue: 10f-THF; Solid Black: MTXPG_1_; Dashed Black: MTXPG_2-7_.

We simulated the effects of differential MTXPG accumulation on the MTX targets in the folate pathway to assess the effects of varying dose and schedule on these targets. We used the previously described enzyme kinetic parameters [12], the MTX and MTXPG inhibition parameters [11], along with the MTX plasma and MTXPG intracellular PK parameters defined in this study. **Figure 6** depicts an individual simulation of the dynamics of the various folate components after infusion of 1 g/m^2^ of MTX over 24 hours. This predicted a two-fold increase in DHF, a one-fold decrease in 5mTHF, and only small changes in the remaining folate components relative to the untreated steady-state levels.

**Figure 7:**
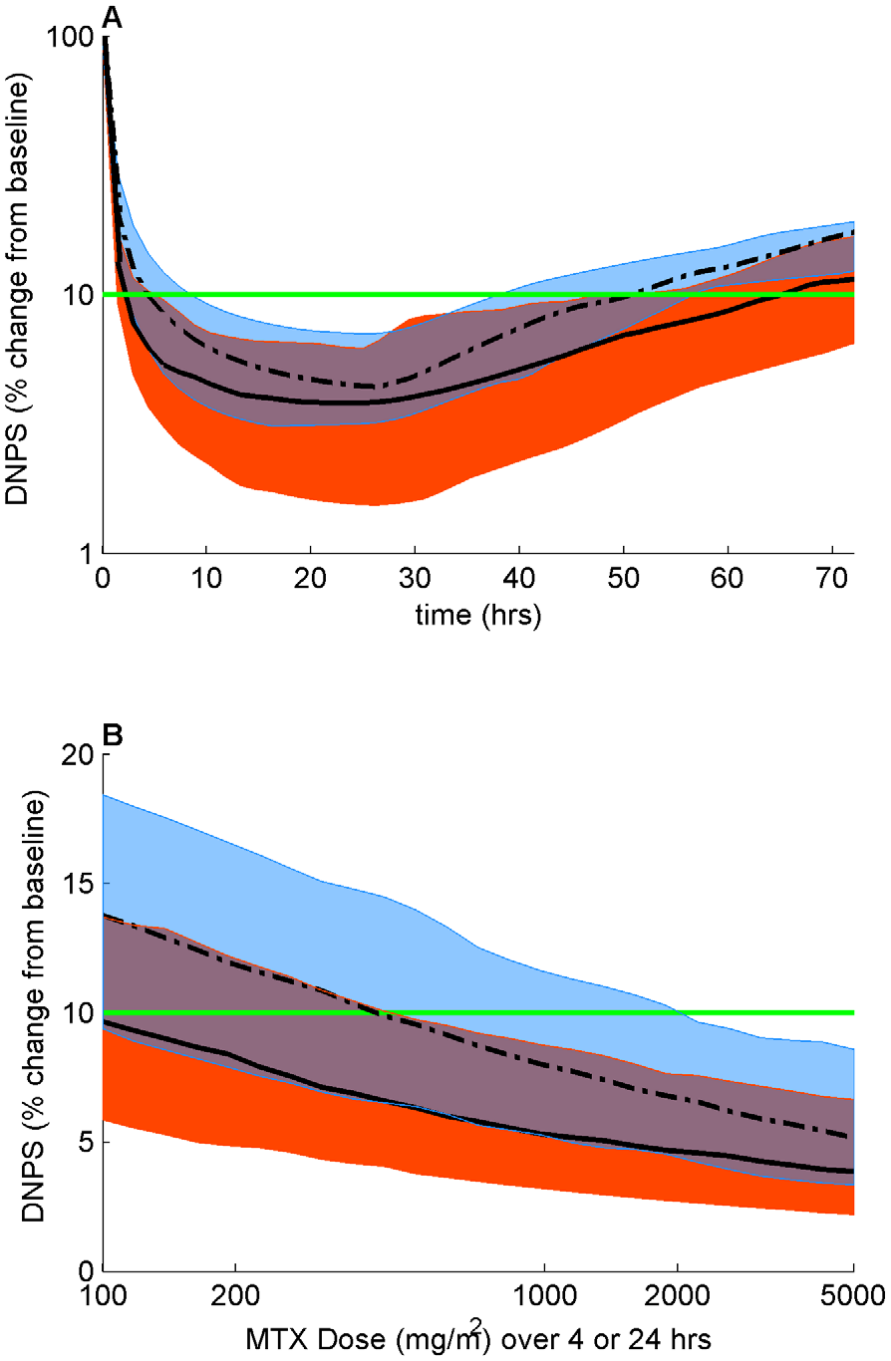
Simulations of the effects of lineage and dosage on model estimated DNPS activity. The curves represent the median and the shaded regions represent the quartiles of the results from the respective simulated patient populations. (**A**) Simulated percent change in DNPS vs time after a 1 g/m^2^ dose of MTX. Solid curve and Red shading: B-lineage Hyperdiploid; Dashed curve and Blue shading: T-lineage. (**B**) Simulated percent change in DNPS at 42 hrs vs Dose. Solid curve and Red shading: 24 hr MTX infusion; Dashed curve and Blue shading: 4 hr MTX infusion.

Because MTXPG accumulation was significantly lower in T-lineage ALL compared to B-lineage hyperdiploid ALL, we investigated how this differential accumulation affected the inhibition of DNPS by comparing the simulated baseline DNPS activity to its activity over a 72 hr post MTX treatment interval. The simulations showed that there was both greater and longer inhibition of DNPS in the B-lineage hyperdiploid group (**Figure 7A**). Next we used simulations to compare the 44 hr post MTX treatment inhibition of DNPS between different doses (100 mg/m^2^ to 5 g/m^2^) and schedules (4 vs 24 hour infusions). As expected, we observed that as we increased dose the simulations predict greater inhibition of DNPS. We also observed greater DNPS inhibition for doses infused over 24 hours compared to 4 hours. Specifically, while a 1 g/m^2^ dose infused over 24 hours was predicted to inhibit about three quarters of the patients' DNPS more than 90%, it was predicted to take approximately a 2.5 g/m^2^ dose infused over 4 hours to produce the same antifolate effects (**Figure 7B**).

## Discussion

MTX is one of the primary anticancer agents used to treat children with ALL and its intracellular accumulation has been shown to relate to its antileukemic effects [4]. The current study allowed us to better understand the basis of differential MTXPG accumulation and how it relates to ALL lineage, ploidy, molecular subtype, gene expression, and genetic polymorphisms. We accomplished this by developing innovative mechanistic pharmacokinetic and pharmacodynamic models of intracellular MTXPG and its interaction with the folate pathway. This gave us a new approach to describing the intracellular disposition of MTXPG (e.g. influx, efflux, FPGS, and GGH activity) along with the effects of MTXPG on the folate pathway. In addition, the model allowed us to easily test hypotheses about which factors have the strongest effects on MTXPG accumulation along with which MTX doses and schedules more effectively inhibits the folate pathway. Specifically, using the pharmacokinetic and pharmacodynamic models, we were able to evaluate a) the mechanisms of intracellular MTXPG accumulation, b) the causes of differential accumulation by lineage, ploidy, and molecular subtype, c) the difference between 4 vs 24 hour MTX infusion (validating simulations in the previous study [9] which showed that longer infusions of MTX at equivalent doses related to higher accumulation of MTXPG), d) the relations between the pharmacokinetic and pharmacodynamic model parameters and mRNA expression of and polymorphisms in and flanking related genes, and e) how MTXPG accumulation affected target enzymes in the folate pathway.

We observed that net influx of MTX was highest in B-lineage hyperdiploid ALL cells which also corresponded to higher RFC expression (SLC19A1). This relation has also been observed in our previous modeling [9] and experimental studies [20]. In addition, we observed differences in the influx and efflux parameters relative to the infusion length of MTX. These differences are most likely attributed to significantly different intracellular disposition of MTXPG_1_ in the 4 hr infusion group compared to the 24 hr infusion group. Specifically, while the population average intracellular concentration of MTXPG_1_ is higher during the first 6 to 8 hours after the start of infusion in the 4 hour group compared to the 24 hr group, the concentrations fall below that of the 24 hour group for the remaining time (**Figure 1B**). These differences could cause an overall increase in the efflux activity for the individuals with higher intracellular concentrations over much of the treatment interval of those in the 24 hr infusion group. Next, we observed differential FPGS activity and net MTXPG accumulation with respect to ALL lineage, ploidy, and molecular subtype. This is concordant with observed differences in FPGS mRNA expression and SNPs in the gene encoding FPGS in both the current study and others [21]–[23].

The folate pathway simulations allowed us to assess the effects of differential MTXPG accumulation on the inhibition of important biosynthetic pathways that are known targets of MTX. One advantage of the modeling and simulation approach was that we could efficiently evaluate multiple situations that would otherwise be difficult, time consuming, and in many cases not practical from a clinical trials perspective. In fact, this is the first time a system of models combining the intracellular disposition of MTXPG and its inhibition of the folate pathway have been used to aid in the understanding of effective MTX therapy. There are two important issues to consider when performing modeling and simulations: the availability of and the sensitivity to the model parameters. Due to the available studies of the folate cycle, there are numerous published estimates of all the primary enzyme kinetic parameters involved (see [12] for a summary). In addition, previous studies have addressed the parameter sensitivity of these folate cycle models by showing that in most cases the effects of changes in the model parameters were local [12]. For example, it was shown that changes in the enzyme kinetic parameters for DHFR had a proportional effect on THF and a much smaller effect on other folates. **Figure S2** shows plots of the effects of changes in V_MAX_ DHFR and V_MAX_ ACAIRT on their respective activities. For these two parameters, only V_max_ACAIRT had a proportional effect on ACAIRT activity and the remaining effects were all minimal. Thus, this effect is considered a local effect. Therefore, the folate model is not sensitive to the parameter choice for V_MAX_DHFR and only locally sensitive to the parameter choice for V_max_ACAIRT.

These simulations helped increase our understanding of how MTXPG accumulation, MTX dose, and MTX schedule affect antileukemic effects. In addition, our simulation results compared qualitatively to previously published studies on MTX inhibition of target enzymes, further validating them. Specifically, the simulation which compared differential accumulation of intracellular MTXPG by ALL lineage showed that in the T-lineage group only about half of the individuals had DNPS inhibition greater than 90% at 44 hrs compared to more than three quarters of the individuals in the B-lineage hyperdiploid group. Also, about half the B-lineage hyperdiploid individuals' DNPS was still inhibited greater than 90% by 72 hours post treatment. These two results are in line with our previously published results [24] which showed that individuals with higher MTXPG accumulation were more likely to achieve full inhibition of DNPS (defined as inhibition greater than 90%). In addition, the simulations describing the effects of MTX dose and schedule showed that there was increased inhibition of DNPS with larger doses and longer infusion schedules. These results are in line with our current clinically measured changes in DNPS in the subset of our patients in which DNPS was directly measured (unpublished data). These results suggest that higher doses of MTX are needed to obtain similar inhibition patterns with shorter (4H) compared to longer (24H) infusions. A recent COG study randomized patients with ALL to receive either a 2 g/m^2^ dose infused for 4 hours or a 1 g/m^2^ dose infused for 24 hours [25]. The results of this study have yet to be reported, but they will provide treatment outcome data that will complement the current study.

In summary, our pharmacokinetic and pharmacodynamic model of plasma MTX, intracellular MTXPG, and the folate cycle provides an important new tool for elucidating mechanisms underlying inter-individual differences in MTXPG intracellular disposition and inhibition of target enzymes. Furthermore, this model permits assessment of how the dosage or schedule of MTX administration alters the delivery of active drug to leukemia cells of different lineage and molecular subtypes. This will facilitate the design of more effective therapy for pediatric ALL.

## Methods

### Patients

A total of 356 patients were enrolled on St. Jude Total XV protocol for newly diagnosed ALL between 2000 and 2007 which stratified and randomized patients to receive MTX during the first day of therapy [26]. This study included the 194 patients who had adequate circulating leukemia cells for intracellular MTXPG quantification at 3 to 4 serial time points during the initial 42 hours of therapy.

### Ethics Statement

The institutional review board approved the study, and informed consent was obtained from parents/guardians or patients. This study was compliant with the regulations of the Health Insurance Portability and Accountability Act of 1996 (HIPAA).

### Treatment Regimen and Sample Collection

Patients were randomized during the first day of therapy to receive either 1 g/m^2^ MTX infused intravenously over 24 or 4 hours. Serial plasma samples were obtained at 1, 4, 24, and 42 hours after the start of the MTX infusion and MTX concentrations were assayed by the Abbottbase TDx-FPIA II assay (Abbott Diagnostics, Irving, TX). In addition, circulating leukemia cells were obtained at 1, 4, 24, and 42 hours after the start of the MTX infusion. Intracellular concentrations of MTXPG were assayed by HPLC as previously described [6], [19].

### Pharmacokinetic Model and Parameter Estimation

**Figure 8:**
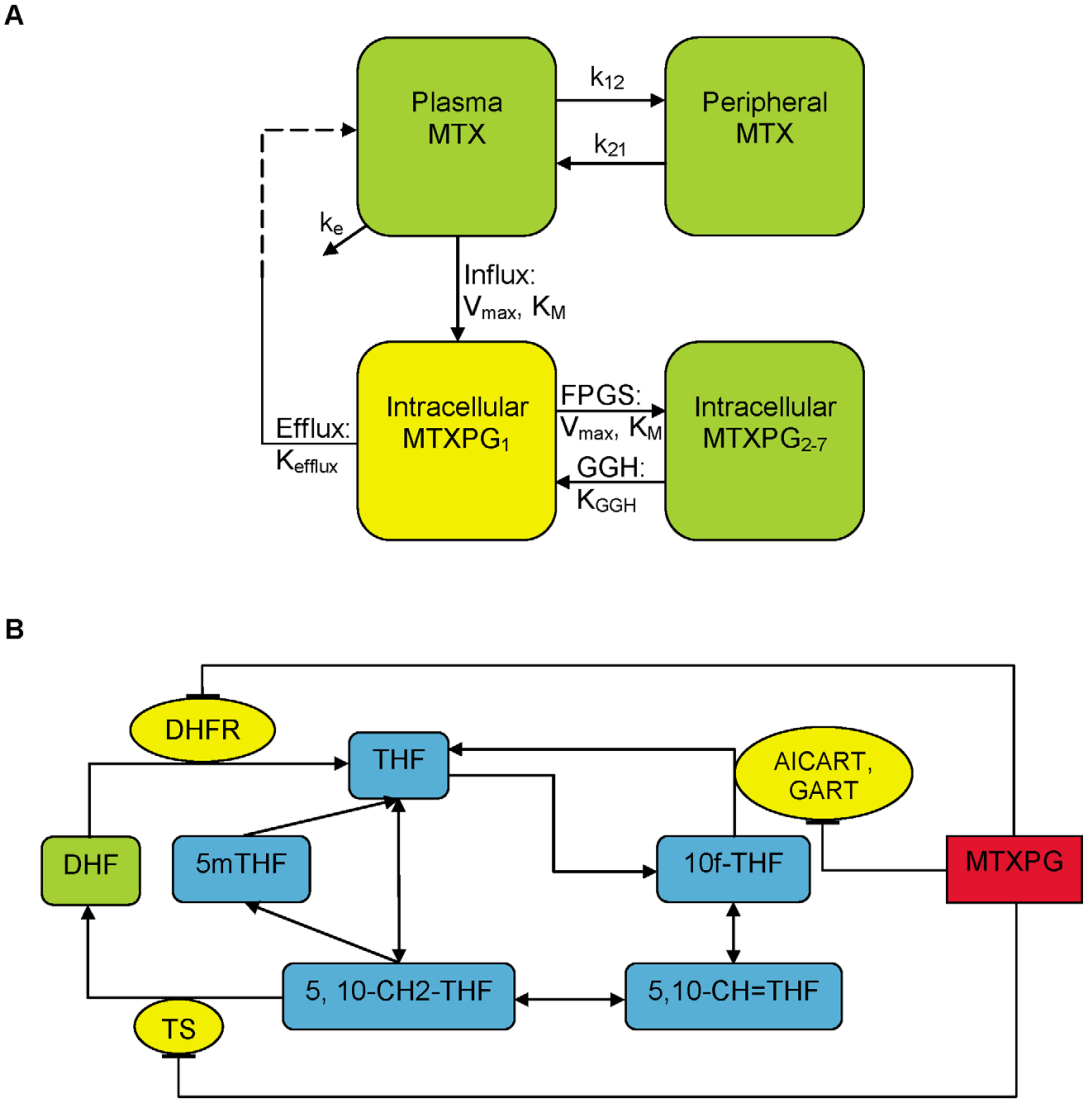
Model schematic. (**A**) The pharmacokinetic model for plasma and intracellular MTX and MTXPG [9]. The parameters are defined in the text. The dashed line describing the efflux of intracellular MTX back to the plasma represents the fact that the system is uncoupled, i.e. we account for the efflux of intracellular MTX but we do not explicitly account for its return to the plasma. (**B**) The folate pathway model [12] is modified to include the pathways inhibited by MTXPG. The model parameters (described by arrows) are defined in the text and supplemental material.

The pharmacokinetic model used to describe the plasma MTX was a first-order two-compartment model (see first two equations in (1)). The pharmacokinetic model to characterize the intracellular disposition of MTXPG was previously described [9]. Briefly, it involves two compartments, one for the intracellular concentration of MTXPG_1_, or intracellular MTX (the third equation in (1)), and the second for the intracellular concentration of MTXPG_2-7_, the sum of MTXPG_2_ through MTXPG_7_ (the fourth equation in (1)), where the subscripts denote the number of glutamates attached to each MTX molecule. A diagram of the model is shown in (**Figure 8A**) and the model is described by the following system of ordinary differential equations:
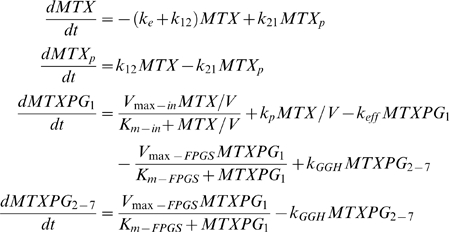
(1)The parameters are defined as follows: *k_e_*, *k_12_*, and *k_21_* (1/hr) are the first-order parameters describing the elimination of plasma MTX and the transition between the central (MTX) and peripheral (MTX_p_) compartments respectively; *V* (L/m^2^) is the systemic volume; *V_max-in_* (pmol/10^9^ cells/hr) and *K_m-in_* (µM) are the Michaelis-Menten parameters describing the active influx of MTX into the leukemic blasts via the reduced folate carrier and various ABC transporters; *k_p_* (1/hr) is the first-order passive influx parameter; *k_eff_* (1/hr) is the first-order efflux parameter; *V_max-FPGS_* (pmol/10^9^ cells/hr) and *K_m-FPGS_* (pmol/10^9^ cells) are the Michaelis-Menten parameters describing the FPGS activity; and *k_GGH_* (1/hr) is the first-order parameter describing the GGH activity. In addition, we defined several secondary parameters which were combinations of the above parameters. These included: Influx (*V_max-in_/K_m-in_*); NET-influx (*V_max-in_/k_eff_*), the ratio of maximum influx activity to efflux—the net influx of MTX; FPGS (*V_max-FPGS_/K_m-FPGS_*); and NET-PG (*V_max-FPGS_/k_GGH_*), the ratio of maximum FPGS to GGH activity—the net accumulation of MTXPG.

We assumed that the amount of drug in the plasma significantly exceeded the intracellular amount. Thus, we did not account for the intracellular drug efflux into the plasma. This allowed us to uncouple the system of four differential equations to two independent systems—one for the plasma pharmacokinetics and the other for the intracellular pharmacokinetics. First we estimated the plasma MTX pharmacokinetics using the maximum *a posteriori* probability (MAP) parameter estimation method implemented in ADAPT 5 [27] along with the prior parameter distribution obtained from previous St. Jude Total protocols [4]. Then, fixing, per individual, these plasma pharmacokinetic parameters, the intracellular MTXPG model parameters (both population estimates and individual conditional means) were determined using the Monte Carlo Parameter Expectation Maximization (MCPEM) [28] with importance sampling population estimation algorithm in ADAPT 5 [27]. This approach was used since, unlike the plasma pharmacokinetics where we had abundant prior parameter information from previous studies, minimal prior information on the distribution of the intracellular MTXPG model parameters was available. Due to the lack of identifiability of the passive influx parameter k_p_ we fixed it to 0.4 (1/hr) —its previously reported value [29]. Finally, due to the known significant differences in the intracellular disposition between B and T-lineage ALL, we fit each lineage group separately in the population model. The individual conditional means were used for comparison to covariates and for the below described folate pathway simulations. The percent relative standard error of the population estimated parameters, as determined in ADAPT 5, was used to assess their sensitivity. In addition, the individual conditional means were estimated ten times using randomly chosen initial parameter values for each run. From these runs the sensitivity of the individual conditional means to changes in initial parameter values was determined by calculating their average relative absolute error.

### Folate Pathway Model

The model used to characterize the folate pathway was taken from Nijhout *et al.* [12] and modified to include the inhibitory effects of MTXPG on target enzymes (**Figure 8B**; equations in **Figure S3**). Specifically, MTXPG was modeled to stoichiometrically inhibit DHFR, TS, and AICART/GART via competitive binding. We simulated the effects of MTXPG on the folate pathway in each patient in the current study by using their respective MTX plasma and intracellular MTXPG model parameters along with published folate pathway enzyme kinetic parameters [11], [12]. We considered simulations over the dose range from 100 mg/m^2^ to 5 g/m^2^ and with a 4 or 24 hr infusion.

### Gene Expression and Polymorphism Methods

Gene expression in ALL cells at diagnosis and germline SNPs in or flanking (within 10,000 bp of the gene) folate transporter (SLCO1B1, SLC19A1, ABCC1, ABCG2) and polyglutamation (FPGS, GGH) genes were determined by Affymetrix HgU133A Human GeneChip arrays and by Affymetrix 500K mapping array genotyping as previously described [5], [30], [31].

### Statistical Methods

Differences in the individual pharmacokinetic model parameters (e.g. the conditional means determined by the above described methods) due to lineage, ploidy, molecular subtype, gene expression, and SNPs were determined by either the Kruskal-Wallis ANOVA or the Mann-Whitney U-test.

## Supporting Information

Figure S1 Representative concentration versus time plots for plasma MTX and intracellular MTXPG model fits. For the MTXPG concentration versus time plots the dashed lines represent the intracellular MTX (or MTXPG_1_) and the solid lines represent the intracellular MTXPG_2-7_ concentration. A) B-lineage Hyperdiploid, 24 hr MTX infusion. B) B-lineage Hyperdiploid, 4 hr MTX infusion. C) B-lineage Non-Hyperdiploid, 24 hr MTX infusion. D) B-lineage Non-Hyperdiploid, 4 hr MTX infusion. E) T-lineage, 24 hr MTX infusion. F) T-lineage, 4 hr MTX infusion.
(0.63 MB PDF)

Figure S2 Sensitivity analysis plots.
(0.47 MB PDF)

Figure S3 Folate pathway model.
(0.06 MB PDF)

Table S1 Summary statistics of all patients randomized in Total XV subdivided by those included in the current study.
(0.07 MB PDF)

Table S2 Population mean parameters for all the patients (n = 194) estimated by Monte Carlo Parameter Expectation Maximization (MCPEM) with importance sampling population estimation algorithm in ADAPT 5. RSE: relative standard error; IIV: Inter-individual variability; CV: coefficient of variation. The Individual Sensitivity Analysis is the median (over the population—n = 194) of the average (over 10 independent estimates each using a different, randomly chosen, set of initial conditions) relative absolute error in the parameter.
(0.06 MB PDF)
