# Supplementary figures and images for: Modeling Mechanisms of In Vivo Variability in Methotrexate Accumulation and Folate Pathway Inhibition in Acute Lymphoblastic Leukemia Cells
Source: PLoS Comput Biol. 2010 Dec 2;6(12):e1001019. doi: 10.1371/journal.pcbi.1001019 (PMC2996318; doi:10.1371/journal.pcbi.1001019)

A)

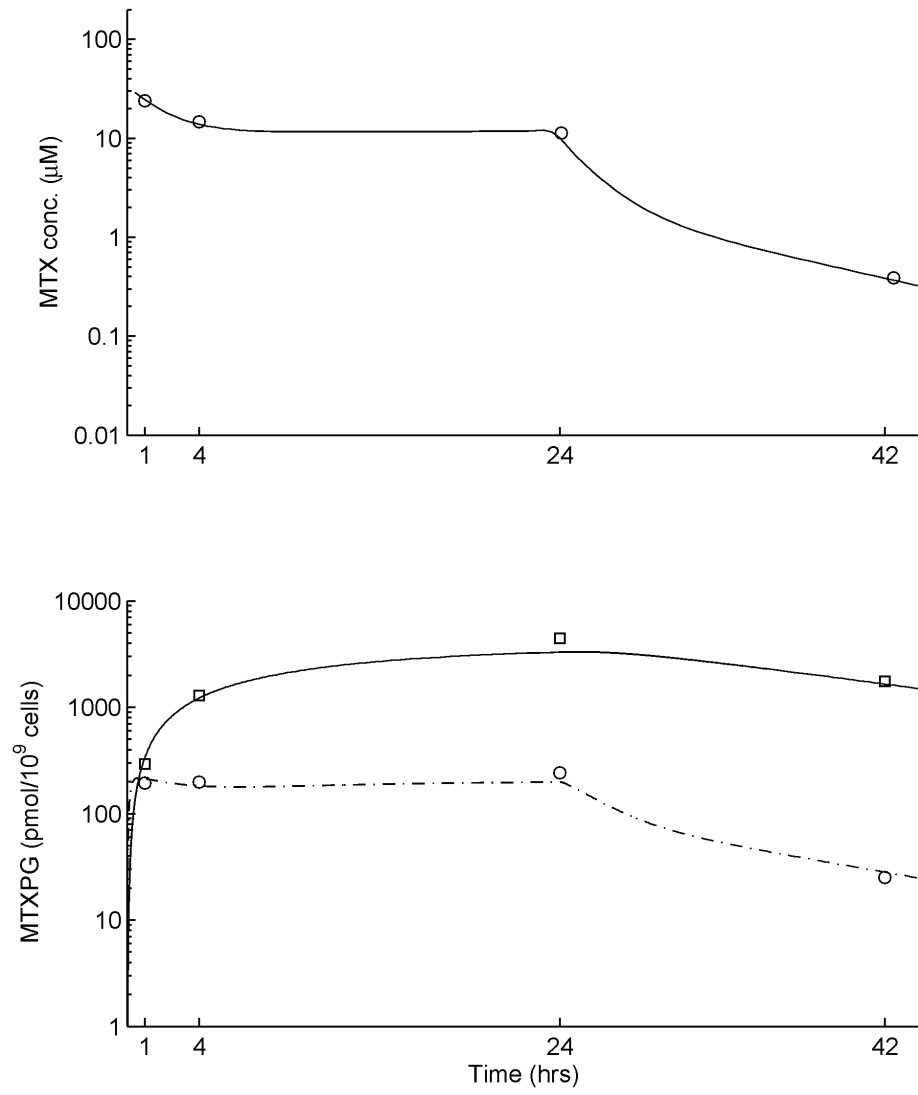

**B)**

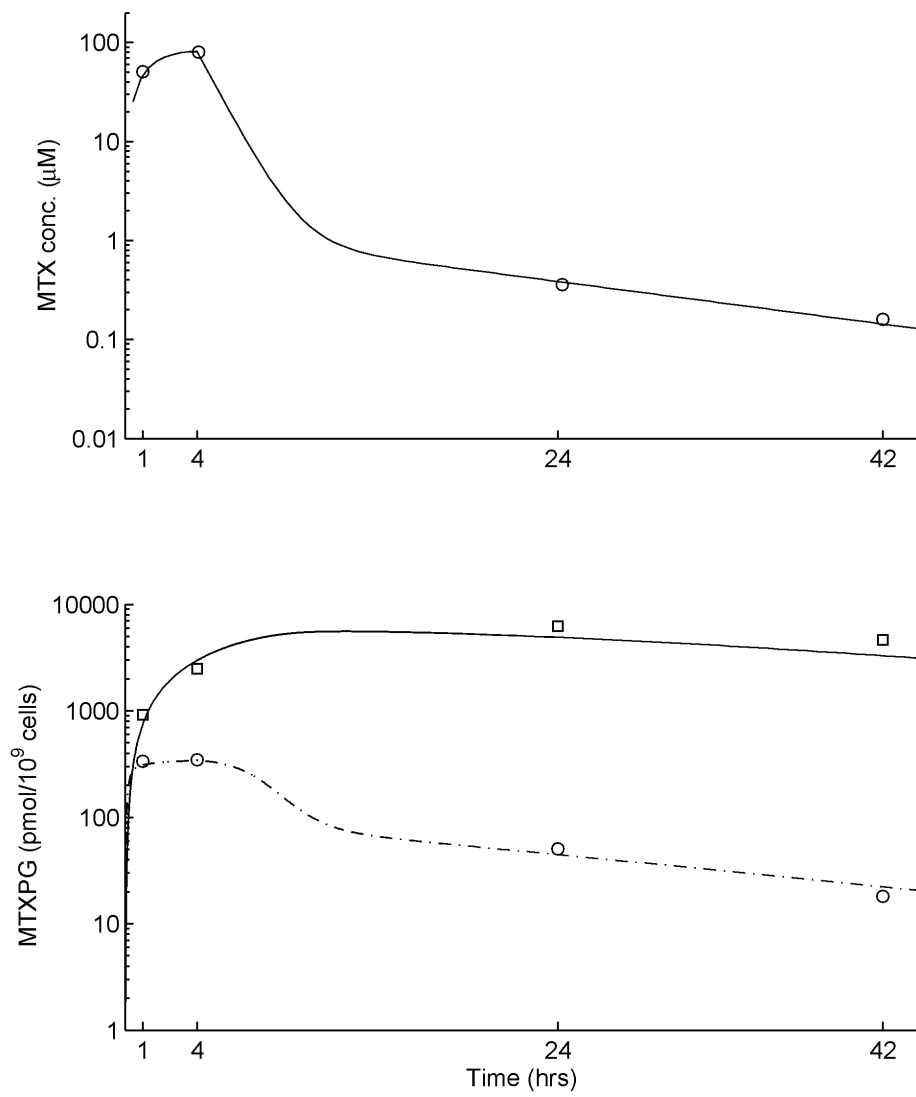

c)

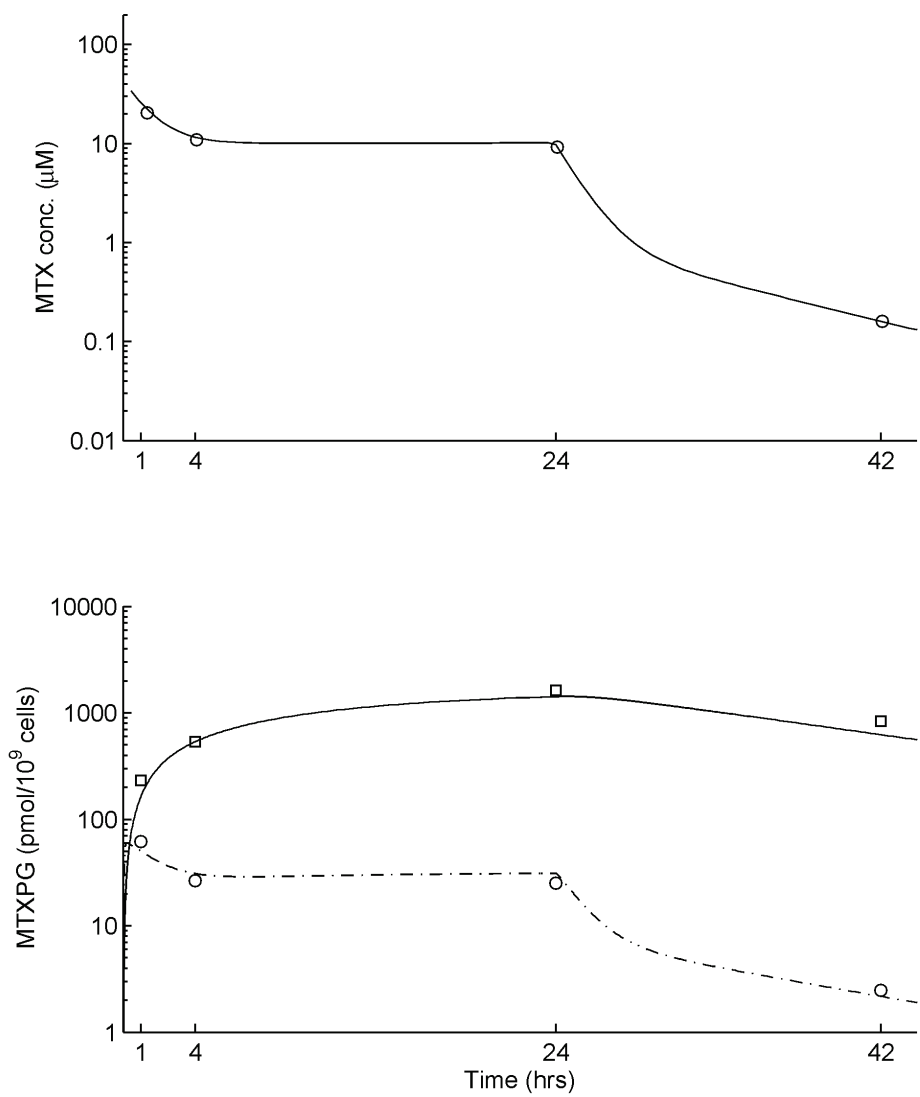

D)

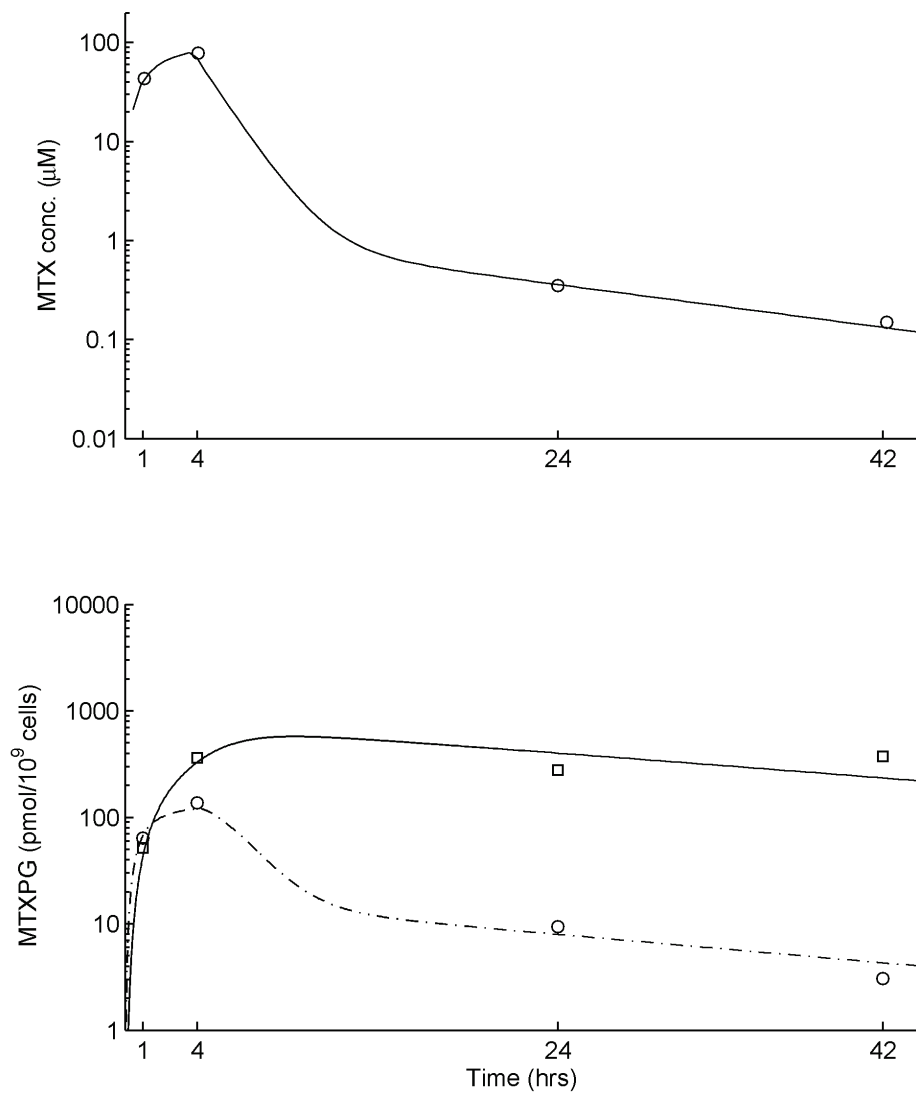

E)

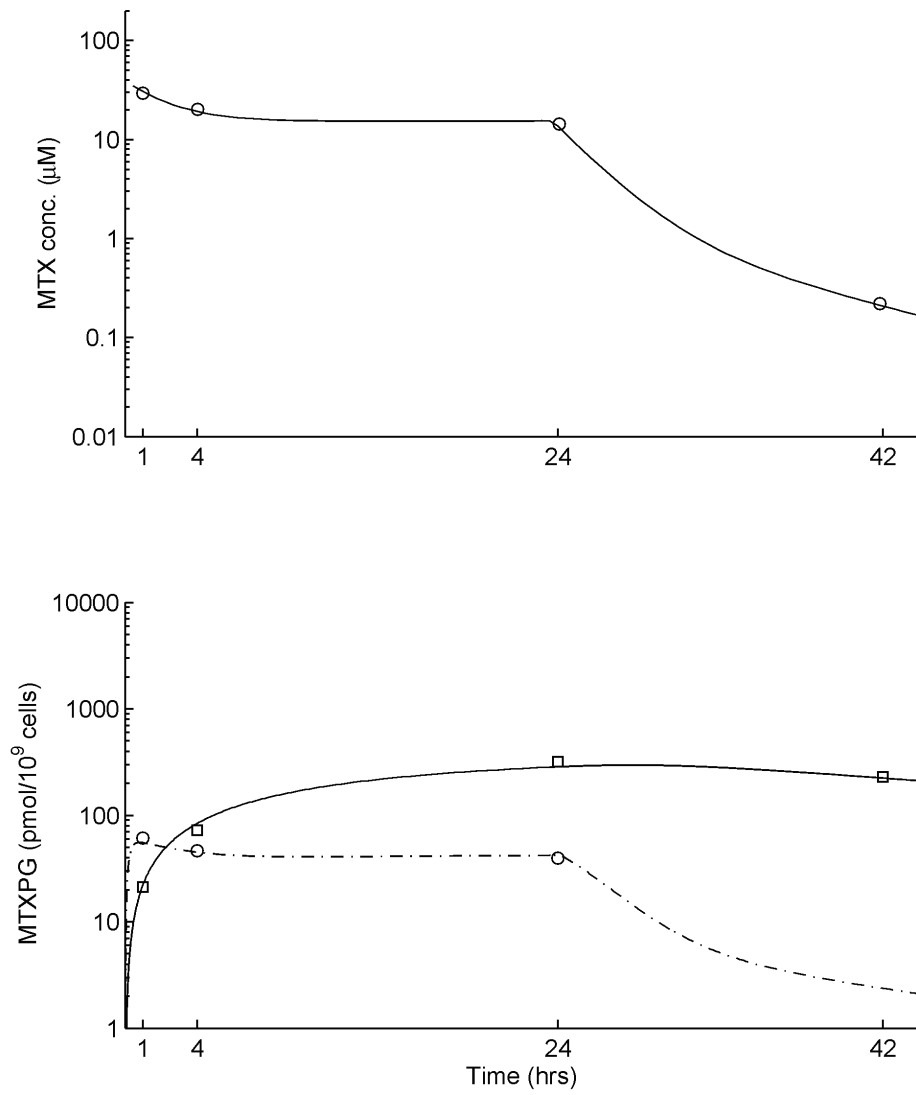

**F)**

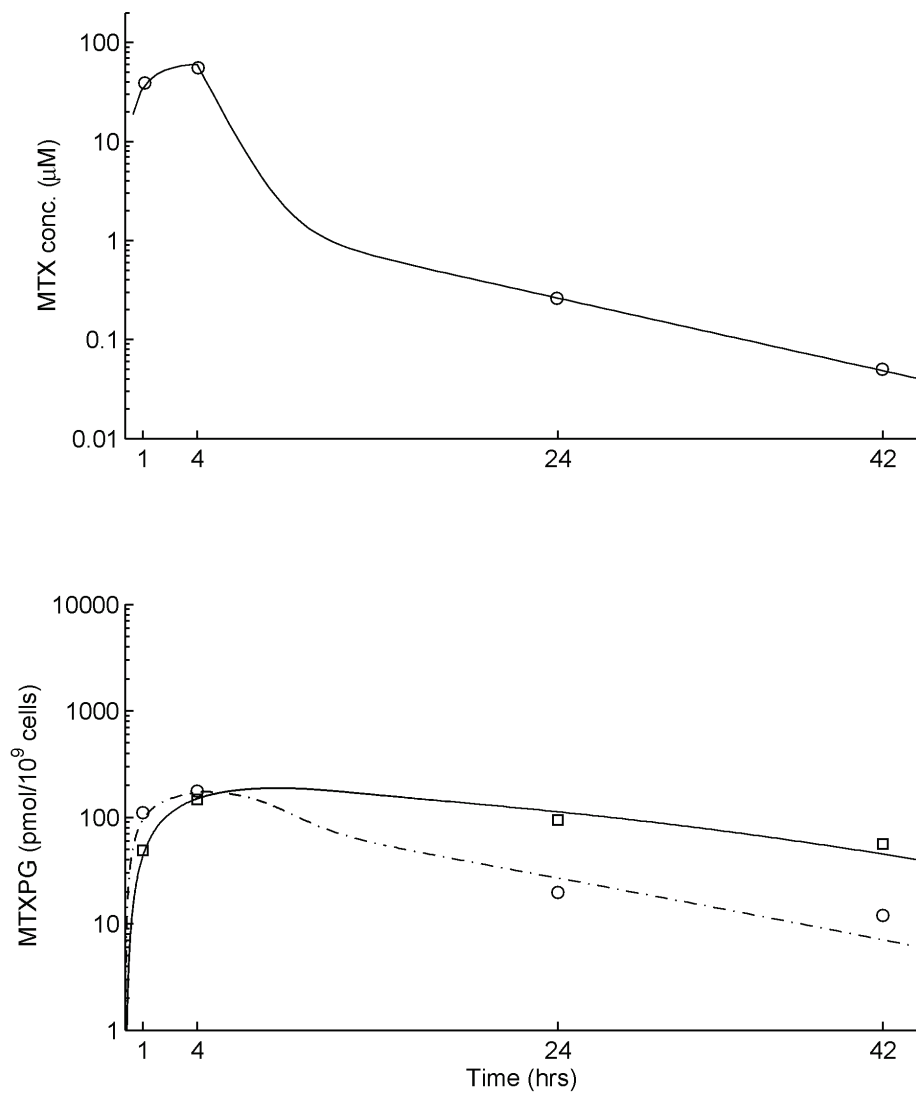

Supplement: Figure S1 — Representative concentration versus time plots for plasma MTX and intracellular MTXPG model fits. For the MTXPG concentration versus time plots the dashed lines represent the intracellular MTX (or MTXPG1) and the solid lines represent the intracellular MTXPG2-7 concentration. A) B-lineage Hyperdiploid, 24 hr MTX infusion. B) B-lineage Hyperdiploid, 4 hr MTX infusion. C) B-lineage Non-Hyperdiploid, 24 hr MTX infusion. D) B-lineage Non-Hyperdiploid, 4 hr MTX infusion. E) T-lineage, 24 hr MTX infusion. F) T-lineage, 4 hr MTX infusion. (0.63 MB PDF) [file pcbi.1001019.s001.pdf]

**A**

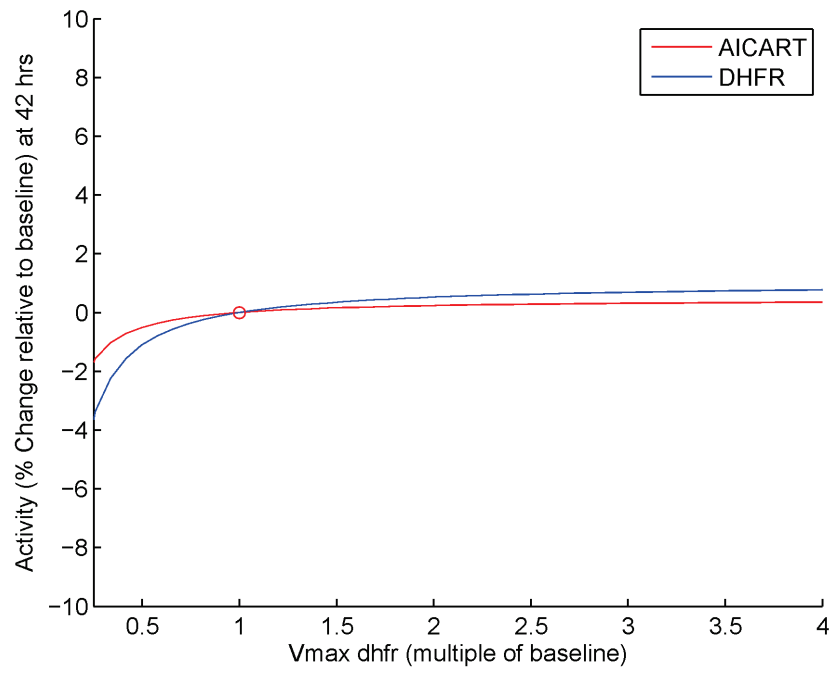

**B**

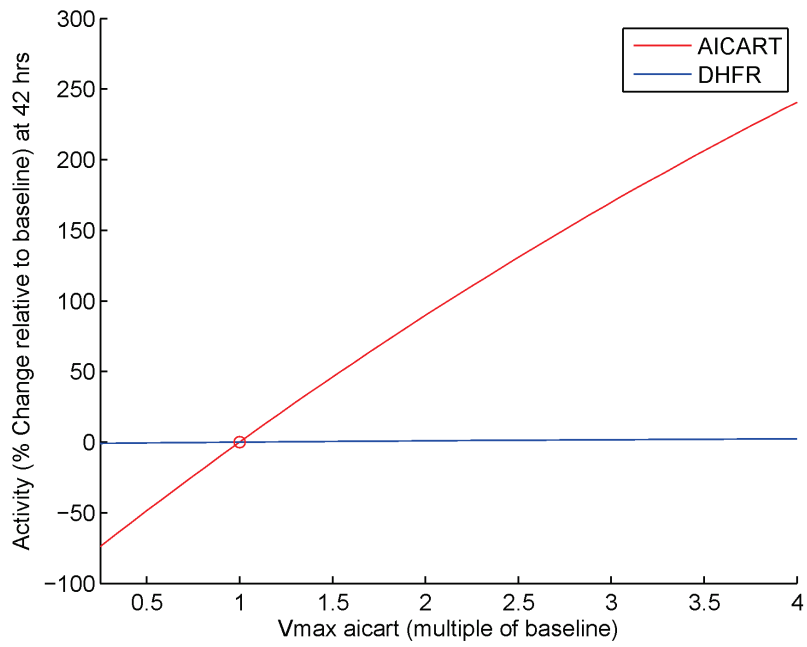

Supplement: Figure S2 — Sensitivity analysis plots. (0.47 MB PDF) [file pcbi.1001019.s002.pdf]
