# Supplementary material for: Modeling Mechanisms of In Vivo Variability in Methotrexate Accumulation and Folate Pathway Inhibition in Acute Lymphoblastic Leukemia Cells
Source: PLoS Comput Biol. 2010 Dec 2;6(12):e1001019. doi: 10.1371/journal.pcbi.1001019 (PMC2996318; doi:10.1371/journal.pcbi.1001019)
Supplement: Figure S3 — Folate pathway model. (0.06 MB PDF) [file pcbi.1001019.s003.pdf]

$$\frac{dDHF}{dt} = V_{TS} - V_{DHFR}$$

$$\frac{dTHF}{dt} = V_{MS} - V_{FTS} + V_{PGT} + V_{AICART} + V_{DHFR} - V_{SHMT} - V_{NE} + V_{FTD}$$

$$\frac{d5,10-CH_2-THF}{dt} = V_{SHMT} + V_{NE} - V_{TS} - V_{MTD} - V_{MTHFR}$$

$$\frac{d5mTHF}{dt} = V_{MTHFR} - V_{MS} + F_{in} - F_{out}$$

$$\frac{d5,10-CH=THF}{dt} = V_{MTD} - V_{MTCH}$$

$$\frac{d10f-THF}{dt} = V_{MTCH} + V_{FTS} - V_{PGT} - V_{AICART} - V_{FTD}$$

Where

$$V_{DHFR} = V_{max,DHFR}\frac{NADPH}{K_{M,NADPH}^{DHFR}+NADPH}\frac{DHF}{K_{M,DHF}\left(1+\frac{MTXPG_1}{K_{I,MTXPG_1}^{DHFR}}+\frac{MTXPG_{27}}{K_{I,MTXPG_{27}}^{DHFR}}\right)+DHF}$$

$$V_{TS} = V_{max,TS}\frac{dUMP}{K_{M,dUMP}+dUMP}\frac{5,10-CH_2-THF}{K_{M,5,10-CH_2-THF}^{TS}\left(1+\frac{DHF}{K_{I,DHF}^{TS}}+\frac{MTXPG_1}{K_{I,MTXPG_1}^{TS}}+\frac{MTXPG_{27}}{K_{I,MTXPG_{27}}^{TS}}\right)+5,10-CH_2-THF}$$

$$V_{MS} = V_{max,MS}\frac{Hcy}{K_{M,Hcy}+Hcy}\frac{5mTHF}{K_{M,5mTHF}+5mTHF}$$

$$V_{FTS} = V_{max,FTS}\frac{HCOOH}{K_{M,HCOOH}+HCOOH}\frac{THF}{K_{M,THF}^{FTS}+THF}$$

$$V_{PGT} = V_{max,PGT}\frac{GAR}{K_{M,GAR}+GAR}\frac{10f-THF}{K_{M,10f-THF}^{PGT}\left(1+\frac{DHF}{K_{I,DHF}^{GAR}}+\frac{MTXPG_1}{K_{I,MTXPG_1}^{GAR}}+\frac{MTXPG_{27}}{K_{I,MTXPG_{27}}^{GAR}}\right)+10f-THF}$$

$$V_{AICART} = V_{max,AICART}\frac{AICAR}{K_{M,AICAR}+AICAR}\frac{10f-THF}{K_{M,10f-THF}^{AICART}\left(1+\frac{DHF}{K_{I,DHF}^{AICAR}}+\frac{MTXPG_1}{K_{I,MTXPG_1}^{AICAR}}+\frac{MTXPG_{27}}{K_{I,MTXPG_{27}}^{AICAR}}\right)+10f-THF}$$

$$V_{SHMT} = V_{max,SHMT}^{SER}\frac{SER}{K_{M,SER}+SER}\frac{THF}{K_{M,THF}^{SER}+THF} - V_{max,SHMT}^{Gly}\frac{Gly}{K_{M,Gly}+Gly}\frac{5,10-CH_2-THF}{K_{M,5,10-CH_2-THF}^{Gly}+5,10-CH_2-THF}$$

$$V_{NE} = k_1 THF - k_2\, 5,10-CH_2-THF$$

$$V_{FTD} = V_{max,FTD}\frac{10f-THF}{K_{M,10f-THF}^{FTD}+10f-THF}$$

$$V_{MTD} = V_{max,MTD}^{pos}\frac{5,10-CH_2-THF}{K_{M,5,10-CH_2-THF}^{MTD}+5,10-CH_2-THF} - V_{max,MTD}^{neg}\frac{5,10-CH=THF}{K_{M,5,10-CH=THF}^{MTD}+5,10-CH=THF}$$

$$V_{MTHFR} = V_{max,MTHFR}\frac{NADPH}{K_{M,NADPH}^{MTHFR}+NADPH}\frac{5,10-CH_2-THF}{K_{M,5,10-CH_2-THF}^{MTHFR}\left(1+\frac{MTXPG_1}{K_{I,MTXPG_1}^{MTHFR}}+\frac{MTXPG_{27}}{K_{I,MTXPG_{27}}^{MTHFR}}\right)+5,10-CH_2-THF}$$

$$V_{MTCH} = V_{max,MTCH}^{pos}\frac{5,10-CH=THF}{K_{M,5,10-CH=THF}^{MTCH}+5,10-CH=THF} - V_{max,MTCH}^{neg}\frac{10f-THF}{K_{M,10f-THF}^{MTCH}+10f-THF}$$

$$F_{out} = \alpha 5mTHF$$
