# Supplementary material for: Modeling Mechanisms of In Vivo Variability in Methotrexate Accumulation and Folate Pathway Inhibition in Acute Lymphoblastic Leukemia Cells
Source: PLoS Comput Biol. 2010 Dec 2;6(12):e1001019. doi: 10.1371/journal.pcbi.1001019 (PMC2996318; doi:10.1371/journal.pcbi.1001019)
Supplement: Table S1 — Summary statistics of all patients randomized in Total XV subdivided by those included in the current study. (0.07 MB PDF) [file pcbi.1001019.s004.pdf]

	Patients in current study	Other patients randomized in Total XV	p-val
Sex, n (percent of total)			>0.08
Female	92 (47%)	62 (38%)	
Male	102 (53%)	100 (62%)	
Self-Declared Race, n (percent of total)			>0.1
Caucasian	154 (79%)	121 (75%)	
African American	30 (15%)	30 (19%)	
Asian	4 (2%)	3 (2%)	
Other	6 (3%)	7 (4%)	
Lineage/Ploidy/Molecular Subtype, n (percent of total)			>0.1
B lineage Hyperdiploid	60 (31%)	46 (29%)	
B lineage Non-Hyperdiploid	52(27%)	56 (35%)	
ETV6-RUNX1 t(12;21)	46 (24%)	31 (19%)	
TCF3-PBX1 t(1;19)	14 (7%)	9 (6%)	
T lineage	22 (11%)	18 (11%)	
Diagnostic WBC ($x10^3$)			$<10^{-6}$
Minimum	2.1	0.4	
1st Quartile	7.0	2.1	
Median	17.3	4.2	
3rd Quartile	61.1	14.3	
Maximum	567	376	
