# Supplementary material for: Modeling Mechanisms of In Vivo Variability in Methotrexate Accumulation and Folate Pathway Inhibition in Acute Lymphoblastic Leukemia Cells
Source: PLoS Comput Biol. 2010 Dec 2;6(12):e1001019. doi: 10.1371/journal.pcbi.1001019 (PMC2996318; doi:10.1371/journal.pcbi.1001019)
Supplement: Table S2 — Population mean parameters for all the patients (n = 194) estimated by Monte Carlo Parameter Expectation Maximization (MCPEM) with importance sampling population estimation algorithm in ADAPT 5. RSE: relative standard error; IIV: Inter-individual variability; CV: coefficient of variation. The Individual Sensitivity Analysis is the median (over the population—n = 194) of the average (over 10 independent estimates each using a different, randomly chosen, set of initial conditions) relative absolute error in the parameter. (0.06 MB PDF) [file pcbi.1001019.s005.pdf]

Parameter	Population Mean	RSE (%)	IIV (CV%)	Individual Sensitivity Analysis (%)
V_{max-in} (pmol/10^9 cells/h)	796.7	11.3	115	13.7
K_{m-in} (μM)	2.46	17.8	221	13.6
K_{efflux} (1/h)	5.48	12.5	132	17.2
$V_{max-fpgs}$ (pmol/10^9 cells/h)	255.8	8.6	94	15.5
K_{m-fpgs} (pmol/10^9 cells)	22.2	16.3	171	27.8
K_{ggh} (1/h)	0.205	10.2	117	6.4
